# The integrative force of political institutions? Direct democracy and voter turnout across ethnic and nativity groups

**DOI:** 10.1186/s40878-020-00216-y

**Published:** 2021-02-22

**Authors:** Anita Manatschal

**Affiliations:** Swiss Forum for Migration and Population Studies SFM, University of Neuchâtel, Rue A.-L. Breguet 2, CH-2000 Neuchâtel, Switzerland

**Keywords:** Direct democracy, Voter turnout, Asian, Hispanic, First- and second-generation immigrants, Political integration

## Abstract

Much has been written on the positive effect of direct democracy (initiatives, referendums) on voter turnout. However, we have limited knowledge about potential differential effects on voters belonging to various ethnic groups. The paper argues that depending on a group’s responsiveness to the political context, direct democracy can (dis-)integrate voters (from) into the electorate. Empirical analysis of Current Population Survey (CPS) voting supplement survey data, together with data on the absolute use of direct democracy across US states, corroborates this theoretical expectation, however lending more support for the disintegrating assumption. Frequent direct democratic elections further widen the negative voting gap between first-generation Asian voters and voters living in the US for three generations or longer, whereas they tend to diminish this voting gap for first-generation Hispanic voters. The disintegrative pattern for first-generation Asian voters remains even significant when excluding California from the state sample, yet not the integrative tendency for first-generation Hispanics. Additional analyses using alternative measures of direct democracy and voting, and applying statistical adjustments to address causality concerns, confirm the robustness of these findings, which shed light on the so-far underexplored (dis-)integrative potential of political institutions.

## Introduction

According to a prominent argument in the literature, direct legislation by means of popular initiatives and referendums inculcates citizens with a sense of civic duty and participatory responsibility, which leads to higher levels of civic engagement and enhances political knowledge and attitudes (Bowler and Donovan 2002; Smith and Tolbert 2007). A more recent revisionist literature challenges this long-term “educative” argument of direct democracy, showing that a frequent use of direct democracy has a short-term mobilizing effect, especially increasing voter turnout – the most fundamental civic activity in a democracy, and a critical component of representation (Childers and Binder 2012; Dyck and Seabrook 2010). However, the literature so far remains silent on potential differential effects across voter groups such as ethnic minority voters, who are often also members of the first or second immigrant generation. Voter turnout varies strongly across ethnic groups, with Hispanics exhibiting higher levels of political mobilization and voter turnout than Asian voters in the US (Lien et al. 2004; Ramirez 2013).

Insights into how political institutions affect minority voting are becoming increasingly relevant. As with other forms of civic engagement, ethnic voting levels are generally lower than those of people whose families have resided in a place for generations (Citrin and Highton 2002; Logan et al. 2012). At the same time, ethnic, racial and immigrant groups constitute the fastest growing minorities in contemporary destination countries. In the 2016 US presidential election, nearly one in three (31%) eligible voters on election day had a Hispanic, black, Asian or other racial or ethnic minority background (Krogstad 2016). There are similar estimates for the first- and second-generation immigrant share of electorates in European countries (Koopmans et al. 2012). In the US, divisions in partisanship and voting between native-born and ethnic, racial or immigrant minorities reportedly outweigh divisions by class, age or gender (Abrajano and Hajnal 2015). Nevertheless, members of ethnic minorities often remain underrepresented in the electoral politics of destination countries (Lien et al. 2004; Logan et al. 2012; Ramakrishnan 2005).

The paper aims thus to scrutinize the (dis-)integrative potential of direct democracy on voting across ethnic groups. To do so, it studies individual voter turnout of the first and second immigrant generation for the two largest ethnic groups in the US – Asians and Hispanics – and compares it to the voter turnout of individuals residing in the country for three generations or longer (hereafter referred to as third-generation-plus).^1^

Connecting institutional theories on the effects of direct democracy with research on immigrant political socialization and mobilization, I argue that ethnic minority voters are, to a varying extent, responsive to the activating effect of frequent direct democratic ballots on voter turnout. While it is clear that whether an individual votes or not is not solely determined by group membership, it nevertheless shapes average individual voting propensity (Lien et al. 2004; Ramirez 2013). I argue that group responsiveness to the activating effect of direct democratic elections should be maximal if 1) voters belong to a group that is familiar with democratic elections, and 2) this group has a high level of non-electoral political mobilization in the destination country.

To empirically study how direct democracy affects voting across ethnic groups, I use individual data (Current Population Survey [CPS] voting supplements from 2002, 2006 and 2010) and a new data set on direct democracy across the 50 US states (Bernauer and Vatter 2019). This allows me to account for the considerable variation of the use of direct democracy (initiatives and referendums) both across states and over time. The selected time frame also allows me to capture a period that was marked by an exceptionally high level of immigrant – especially Hispanic – political mobilization (Pantoja et al. 2001; Ramirez 2013; Zepeda-Millán 2017). Based on their group-level familiarity with democratic elections, and degree of non-electoral political mobilization at the time, I expect Hispanics to be most responsive, and Asians to be least responsive, to the activating effect of direct democracy.

The empirical results substantiate differential effects, lending however stronger support for a disintegrating function of direct democratic elections. A frequent use of direct democratic instruments during “normal” electoral times (i.e. midterm elections which are not distorted by presidential campaigns) narrows down the negative voter gap between third-generation-plus voters and first-generation Hispanic voters, but widens this gap even further for first-generation Asian voters. Additional analyses excluding the case of California – the state with the most frequent use of direct democracy and very high shares of Hispanics and Asians in the electorate – reveal that even in this very conservative robustness check the disintegrating pattern for first-generation Asians remains significant, yet not the integrative tendency for first-generation Hispanic voters. No differential effects emerge when comparing second-generation Asian or Hispanic voters to third-generation-plus voters. Additional robustness tests, including alternative measures of direct democracy (content of direct democratic ballots, mere institution of direct democracy) and voting behavior (presidential elections) as well as statistical adjustments to address causality concerns, corroborate these main results.

The study adds to the body of research on the effects of direct democracy on civic engagement by theorizing and empirically substantiating group differential effects of the frequent use of direct democracy on individual voter turnout. By focusing on differential effects, the paper puts unequal electoral participation at its core, challenging the premise that democratic systems entail equal participation (Dahl 1989).

Second, with its focus on general political institutions, the study complements research on the political behavior of ethnic and/or immigrant minorities, which rarely goes beyond immigrant-specific political institutions or policy frameworks, such as naturalization, integration or non-citizen enfranchisement policies (Bloemraad 2006; Filindra and Manatschal 2019; Vernby 2013). If the majoritarian political institution of direct democracy is investigated in this context, studies generally focus on its inherent risk of becoming a tyranny of the majority over the immigrant or ethnic minority (Manatschal and Bernauer 2016; Vatter et al. 2014). In the US context, we find a rich literature documenting how nativist legislative proposals mobilized immigrants in general, and Hispanics in particular, via threat (Pantoja et al. 2008; Zepeda-Millán 2017). This is a narrow perspective since immigrants are, of course, not only exposed to immigrant-specific policies and institutions, but to the entire political system.

Third, the study corroborates the need to formulate context- and time-specific theories in migration research in order to do justice to the high degree of complexity, heterogeneity, as well as the dynamism that typically characterizes migration-related research topics (Castles 2010). The present paper exemplifies how this can be done by formulating and testing theoretical expectations about differential effects of direct democracy on voters from various ethnic groups in a period of intense immigrant political mobilization.

## Theoretical background

### Direct democracy and voter turnout

Classic democratic theory argues that direct democracy is not just of value in and of itself, but that it also has an educative role that promotes a sense of efficacy and civic engagement (Barber 1984; Bowler and Donovan 2002, 374). It is based on the expectation that direct legislation inculcates citizens with a sense of civic duty and participatory responsibility, thereby increasing voter mobilization (Bowler and Donovan 2004, 345; Smith and Tolbert 2007). Furthermore, associations play a crucial role for collective action in direct democratic systems, either to influence government through public pressure, or to address needs, threats and conflicts in the absence of a government response (Freitag 2006). Combining these arguments, direct democracy is assumed to strengthen the civil society sector, voluntary activities, and political activism and engagement, as it delegates responsibility to the citizen rather than to the state and government (Freitag 2006; Smith and Tolbert 2007). Subnational comparative research on Switzerland and the US, the two countries with the most extensive instruments and use of direct democracy at the subnational level (Matsusaka 2004, ix), offers abundant empirical evidence on the participation-enhancing effect of direct democracy on civic and political engagement, ranging from informal volunteering activities to the formal electoral process of voting (Biggers 2011; Bowler and Donovan 2002; Freitag 2006; Smith and Tolbert 2007; Stadelmann-Steffen and Freitag 2011).^2^

A more recent revisionist literature challenges and refines this classical “educative” argument of direct democracy (Stadelmann-Steffen and Freitag 2011). Studies focusing mainly on direct democracy in the US show that voter turnout is not increased by a long-term educative effect, but rather by short-term mobilization through political campaigns and current direct democratic votes, with some carryover from previous elections (Childers and Binder 2012; Dyck and Seabrook 2010). The extent of mobilization via direct democratic elections also depends on the issues at stake. Research shows that especially contested social issues, e.g. morally charged topics including abortion, same sex marriage, or drug legalization, which typically generate a strong moral response by citizens as they touch upon core values which are rooted within a citizen’s belief system, mobilize turnout (Biggers 2011, 2014).

With respect to the type of direct democratic instrument, Childers and Binder (2016) suggest that referenda are not inherently different than initiatives and that both campaigns can mobilize voters. Research further shows that this mobilizing effect on voter turnout is most prominent in midterm elections, where state campaigns do not have to compete with presidential campaigns and are therefore more likely to successfully connect with voters (Childers and Binder 2012; Smith and Tolbert 2007).^3^ However, research on differential outcomes of direct democracy for particular groups is scarce. One notable exception is the study by Hero and Tolbert (2004), which finds that frequent exposure to direct democracy has no detrimental impact on racial and ethnic group attitudes toward government.

According to a competing argument, frequent direct-democratic elections can also have the opposite effect and deter citizens from voting. So far, empirical evidence for this argument has mainly been presented for the Swiss case, where scholars refer to voter fatigue and the high complexity of Swiss political institutions to explain the deterring effect of frequent ballots (Blais 2014; Freitag and Stadelmann-Steffen 2010). This finding clearly contrasts with the enhancing effect of referendums and initiatives on turnout documented above for American States. The stimulating effect of direct democracy is further highest when decisions on ballot measures are concurrent with elections (Freitag and Stadelmann-Steffen 2010; Geys 2006), which is typically the case in the US, but not in Switzerland.

Up to this point, research has not differentiated the extent to which the activating effect of a frequent use of direct democracy extends to various groups of the citizenry. Voters belonging to some societal groups may be more (or less) activated by direct democracy than others. A possible integrative mechanism would be particularly relevant for minority voters, given that citizens with an ethnic background are underrepresented in electoral politics.

Summing up the revisionist literature, we can however draw the following lessons for the present study on differential effects of direct democracy on various ethnic groups: What seems to matter most for increasing voter turnout in “normal” electoral times (midterm elections) is the actual and current use of direct democracy in terms of initiatives and referendums (Childers and Binder 2012; Smith and Tolbert 2007). Voter turnout does not seem to be altered by the mere institutional provision of direct democratic instruments per se, nor by their long-term use (Bowler and Donovan 2002; Hero and Tolbert 2004; Smith and Tolbert 2007). Where initiatives appear frequently on state ballots, active campaigns or media coverage may focus public attention on major public issues, and public discussion and voter mobilization are likely to follow (Bowler and Donovan 2002, p. 377). An important, yet implicit, precondition for the activating effect of direct democracy is therefore that voters are responsive to the political context surrounding them.

### Ethnic voter turnout

Research scrutinizing political participation highlights a variety of reasons for lower voter turnout among voters with an ethnic and/or immigrant background. The standard individual model argues that political participation requires resources in terms of time, political experience, information, education, money and knowledge (Verba et al. 1993). Research further highlights the relevance of local rootedness – in terms of residential stability, house-ownership or marriage – for ethnic political participation (Ramakrishnan and Espenshade 2001). Lower participation rates by Latinos and Asians were traditionally explained through differences in the resources and rootedness of community members (Leighley and Vedlitz 1999; Ramakrishnan and Espenshade 2001; Ramakrishnan 2005). For instance, Citrin and Highton (2002) attribute low Hispanic voting rates to Latinos’ lower share among naturalized citizens, relative youth and lower socioeconomic status. However, the massive immigrant, and especially Hispanic, mobilizations throughout the late 1990s and early 2000s led to a surge in naturalizations and a steep increase in voter turnout among Latinos (Barreto et al. 2009; Pantoja et al. 2001; Ramirez 2013), challenging the classical resource model of electoral participation. Asian-American citizens’ depressed voting rates remain puzzling, given their elevated socioeconomic position (Logan et al. 2012). Several studies have shown that despite their resources, eligible Asian Americans participate and vote at disproportionately lower rates than other groups (Lien et al. 2004; Wong et al. 2011). Less than half of Asian American citizens voted in the 2016 presidential election (Masuoka et al. 2018).

A similarly diverse picture emerges for different nativity groups. In line with assimilation theory (Alba and Nee 2003), one would expect recent immigrants to be slowly incorporated into mainstream society. Given their economic, cultural, residential and linguistic assimilation, the second and later generations should progressively approximate native-born voter turnout levels (Logan et al. 2012). While some researchers show that foreign-born individuals are indeed less likely to vote than second-generation immigrants (Cho 1999; DeSipio 1996), others point to mixed evidence (Logan et al. 2012; Ramakrishnan and Espenshade 2001). Considering the effect of national identification on turnout (Huddy and Khatib 2007), one could for instance argue that individuals from the first immigrant generation who decide to go through the burdensome process of naturalization feel closer to the country, which may reflect in higher political participation compared to the second generation, who automatically acquired US citizenship at birth.

Since individual resources or rootedness cannot entirely account for group differences in voter turnout, scholars increasingly investigate the relevance of collective or contextual factors related to the reception context. As Cho (1999) points out, it is not so much higher education or social status per se which explains ethnic minority voting, but the socialization process that is associated with these factors; this involves exposure to and embracing the norms of a given political system. Related to this point, research highlights the importance of accounting for group contexts when studying the electoral participation of ethnic, racial or immigrant minorities (Logan et al. 2012).

### Group responsiveness to the political context

To theorize and assess differential effects of direct democracy on individual voting across ethnic groups, we must therefore account for varying levels of group responsiveness to the political system. In this study, responsiveness implies that individual voters receive information on upcoming direct democratic elections, for example via active campaigns, media coverage, or discussion with colleagues and friends. It also implies that individual voters are able to develop an informed opinion about the issues at stake, which may in turn motivate them to vote. Given the quantitative nature of this study, it is not possible to trace back individual political opinion-forming and mobilization processes. However, it can identify factors relating to the ethnic and immigrant background of individual voters, which make it more or less likely that an average individual of a specific group responds to the mobilizing effect of direct democracy. I expect two factors to be decisive in shaping individual responsiveness to direct democratic elections: an ethnic group’s average degree of familiarity with democratic elections and of non-electoral political mobilization.

Confirming the political socialization literature (Greenstein 1965), research documents that the formative years in early childhood are decisive for immigrant political behavior in later stages of life (Li and Jones 2019). In the case of first-generation immigrant voters, this implies that they carry over the political socialization of their home country, which affects their political behavior in the new destination country (Bloemraad 2006; Cho 1999; Wass et al. 2015). If an immigrant originates from a democratic country, she is, for instance, more familiar with the practice of voting compared to a person who was born in an autocratic state.

Empirical evidence arising from the CPS survey data used in this study reveals that in the 2010 midterm elections, more than 52% of first-generation Asian-American voters originated from a non-democratic (meaning hybrid or autocratic) regime.^4^ If we include the borderline case of the Philippines, the share of non-democratic states among Asian countries of origin increases to 70%. In contrast, only 8% of first-generation Hispanic voters in the CPS sample from 2010 come from a non-democratic Latin American country. Accordingly, I expect voters coming from Latin America to exhibit, on average, a similar degree of familiarity with democratic elections and the process of voting when compared to the reference category of third-generation-plus voters (see Table 1). Conversely, I expect first-generation Asian-American voters’ average experience with democratic elections to be lower when compared to third-generation-plus voters. Turning to second-generation voters, they should exhibit similar levels of experience with democratic elections as third-generation-plus voters, irrespective of ethnic background, since they were born in the US.

**Table 1:** Explanatory model for group responsiveness to direct democratic elections

	3rd generation plus	2nd generation		1st generation	
		Asian	Hispanic	Asian	Hispanic
**Experience with democratic elections**	Ref. cat.	similar	similar	lower	similar
**Non-electoral political mobilization**	Ref. cat.	lower	higher	lower	higher
***Activating effect*** **via** ***DD compared to 3rd generation plus***	*Ref. cat.*	*same or lower*	*same or higher*	*lower*	*same or higher*

*DD* Direct democracy

With respect to non-electoral political mobilization in the US, I expect individuals who belong to ethnic groups with higher non-electoral political mobilization to be more responsive to the political context in general, and to the activating effect of frequent direct democratic elections in particular. A high level of non-electoral political mobilization implies a high level of organizational density and social networks that connect individual voters with the political system. These organizations and networks play an important role in informing individuals about upcoming direct democratic elections, fostering individuals’ civic and political skills, and eventually also in mobilizing individuals to turn out to vote (Bowler and Donovan 2002; Freitag 2006).

The early 2000s, the time period analyzed here, is a particularly interesting period in this regard. It was shaped by massive mobilizations and protests, especially by Hispanic immigrants and their co-ethnics, in response to restrictive immigration policies at the national or state level, and the perceived threat emanating from these policies (Pantoja et al. 2001; Ramirez 2013; Zepeda-Millán 2017). Scholarly attention has focused on the mobilizing effect of anti-immigrant policy proposals such as California’s Proposition 187 in 1994 and the so-called “Sensenbrenner Bill” HR 4437 in 2005. While neither of these policies were enacted in the end, political discussions around these and other nativist policy proposals in various US states spread a climate of anti-immigrant threat leading to the largest immigrant (especially Hispanic) mobilizations in the US to date.

Research on the particular case of Proposition 187 in California, which sought to deprive undocumented immigrants of access to public services, shows that the proposal was widely perceived as an anti-Hispanic vote, which mobilized Hispanics in and beyond California (Bloemraad et al. 2011; Garcia Bedolla 2005; Ramakrishnan 2005, p. 117). Among Hispanics especially, the effects on immigrant political behavior were comprehensive; they included mass protests, a rise in naturalization rates, increased attention to politics, an increase in voter turnout and a voter shift toward the Democratic Party (Barreto et al. 2009; Bowler et al. 2006; Pantoja et al. 2001; Ramakrishnan 2005). Asian immigrants, on the other hand, did not sense the same degree of threat over Proposition 187, indicating a clear racial bifurcation in political mobilization (Ramakrishnan 2005, p. 130). California’s large and diverse Asian community also supported the anti-proposition movement, and there is evidence of defensive naturalization among Chinese immigrants during this time, however this phenomenon remains restricted to large West Coast cities (Ong 2011). Overall, Asian mobilization was relatively small (Barreto et al. 2009).

We observe a similar pattern of non-electoral mobilization at the national scale in the early 2000s, the period under study here, especially in the context of the encompassing immigrant response to the threat emanating from HR 4437, a 2005 bill that would not only have criminalized illegal presence in the US but would also have punished anyone who aided these immigrants (Bloemraad et al. 2011; Zepeda-Millán 2017). Again, HR 4437 represented a powerful external threat that particularly activated Hispanics, thereby creating a broad Hispanic movement that was national in scope and cut across generations, countries of origin and legal statuses (Barreto et al. 2009). “Today We March, Tomorrow We Vote” was one of the common slogans helping to activate Hispanics (Zepeda-Millán 2017, 176ff). The nativist policy threat of the time had a unifying effect as it activated multiple Latino constituencies to unite in group solidarity, thereby reinforcing the emergence of a pan-ethnic Latino identity (Barreto et al. 2009; Pantoja et al. 2008; Ramirez 2013; Zepeda-Millán 2017). The 2006 mobilization of young immigrants, many of them from the second generation, was remarkable (Pantoja et al. 2008). Similar to the events of the late 1990s, there was a Latino-Asian alliance over nativist policy. Although Asian Americans marched in every city across the country, their turnout was significantly lower than that of Hispanics (Barreto et al. 2009). What is more, Asian American group consciousness continues to be more dynamic and layered than Hispanic group identity; Asian Americans across generations simultaneously continue to identify with their heterogeneous national-origin identities such as Korean American, Indian American or Japanese American (Lien et al. 2004; Wong et al. 2011).

As illustrated in Table 1, the political events of the early 2000s suggest that Hispanics of the first and second generation experienced a very strong political mobilization via threat. During that time, Hispanic group mobilization via non-electoral participation exceeded non-electoral political mobilization among third-generation-plus voters. Conversely, non-electoral political mobilization among Asian voters was limited, remaining behind the mobilization levels of third-generation-plus voters.

Connecting the assumptions about familiarity with democratic elections with the observations regarding group-specific political mobilization at the time, I therefore expect the following group differential effects of direct democracy on individual voter turnout: Given their exceptionally high degree of non-electoral political mobilization and familiarity with democratic elections, I expect Hispanic voters of the first and second generation to be most responsive to the activating effect of direct democracy. In other words, at the time of study in the early 2000s, direct democracy may even have had an integrative function, narrowing the negative participation gap between first– and second-generation Hispanic voters, compared to third-generation-plus voters. Due to their lower levels of non-electoral political mobilization, I expect Asian voters in turn to be less responsive to the mobilizing effect of direct democratic elections than third-generation-plus voters. This disintegrating effect of direct democracy on Asian voter turnout should be particularly salient among first-generation Asian voters, who are on average less experienced with democratic elections.

## Data and research design

The empirical analysis on how the use of direct democracy in the US relates to individual voter turnout across ethnic and nativity groups relies on survey data from the voting supplements of the CPS, as well as a fresh data set on the existence and use of direct democratic instruments in US states (Bernauer and Vatter 2019). CPS offers several advantages for the study of voting across immigrant generations, as it is a nationally representative survey including detailed information on the voting and nativity status of the respondents and their parents. As the CPS voting supplement is collected every November of congressional and presidential election years, it is the only survey that has consistently collected information on voting, ethnic background, and generational status for the election years being studied.^5^ The dependent variable is a dummy, capturing whether respondents did or did not vote in the midterm elections of 2002, 2006, and 2010. In the sample of midterm election voters and non-voters, 212′599 respondents belong to the reference category of third-generation-plus voters. 5′766 are first- and 4′860 s-generation Hispanics. 4′585 respondents indicated an Asian background of the first, and 1′735 of the second generation.

Using CPS data to analyze voting participation also implies certain limitations. Apart from the inferential challenges of survey data, which I will discuss further below, CPS does not for instance include any individual-level information on political attitudes or party contact, which are important predictors of voting behavior (Ramakrishnan and Espenshade 2001, p. 880). However, since the focus of this study is on political institutions and voter turnout, and not on political attitudes or other aspects of political behavior, this limitation seems acceptable. Another limitation of the CPS relates to vote misreporting, although it has been shown to be considerably lower in the CPS than in other surveys such as the National Election Study (Ramakrishnan 2005).

To measure the provision and use of direct democratic instruments in the 50 US states, I use a new data set including information on whether the following five direct democratic instruments exist, and if so, how often they have been used in a given state: 1) statutory or 2) constitutional popular initiative, 3) statutory or 4) constitutional legislative referendum, and 5) popular referendum (for an overview see Donovan 2014, Lupia and Matsusaka 2004). The main independent variable is a count variable capturing how often the five aforementioned direct democratic instruments have been used in a given state in the 2 years preceding the respective midterm elections. This variable fluctuates considerably over time; for example, direct democratic instruments were only used once in the state of Maine in the 2 years up to 2002, compared to eight times in the 2 years up to 2010. Figure OA1 in the online appendix illustrates also pronounced cross-state variance. It shows the yearly average use of direct democracy in the 50 US states between 2000 and 2010, which ranges from 0 (Delaware) to an average of 9.6 direct democratic elections per year in California. As elaborated in the theoretical section, and in line with the existing literature, I expect that voter activation through direct democracy works primarily through exposure to recent direct democratic elections, accounting for both popular initiatives and referendums.

In accordance with the literature on civic engagement in general, and on voting in particular, the analyses further control for individual socioeconomic and socio-demographic variables such as age, gender, education, marital status and labor force participation (Brady et al. 1995; Cho 1999; Ramakrishnan 2005). Racial background is an explanatory factor of black voter mobilization and is controlled for in this paper. To test the expected differential effects of direct democracy, effects on individual voter turnout will be compared across ethnic groups (first- and second-generation Hispanic and Asian citizens) as compared to third-generation-plus voters. Besides direct democracy, controls at the state level include state political ideology, organizational density as a measure for the civic culture of a state, economic performance (unemployment, GDP), degree of urbanization and foreign-born share. For more details on all variables used in the analysis, descriptive statistics and data sources see Table OA1 in the online appendix.

Methodologically, the empirical analysis examines the relationship between direct democracy and individual voter turnout across the 50 US states at three points in time – the midterm elections of 2002, 2006, and 2010 – and using logistic regression analyses. The three time points are pooled, resulting in a data structure with 150 observations at the macro level of states and years. Unobserved heterogeneity across states and years will be accounted for using state and year fixed effects.^6^

## Empirical results

The empirical test of the theoretically expected differential effects of direct democracy proceeds in three steps. In the first step, first- and second-generation Asian and Hispanic voters are compared to third-generation-plus voters, in order to substantiate the preliminary theoretical assumption of voter gaps between groups. In step two, the use of direct democracy is added to the analysis and interacted with the different ethnic group characteristics to test the postulated moderating effects of direct democracy on these voter gaps. Step three discusses a comprehensive series of additional analyses testing the robustness of the main findings.

### Individual voter profiles and voter gaps between ethnic and nativity groups

The first set of logistic regressions on individual voter turnout substantiates the expected voter gaps across ethnic and nativity groups. As Models 1 and 2 in Table 2 confirm, this gap is largest for first-generation Asian and Hispanic voters, as they have the lowest voting probability when compared to third-generation-plus voters. Furthermore, Models 1–2 in Table 2 corroborate the theoretical acculturation argument, according to which the negative voting gap relative to the third-generation-plus reference category gets smaller the longer an individual lives in a country. In both models reported in Table 2, the negative coefficient relative to third-generation-plus voters is smaller for the second than for the first generation.

**Table 2:** Logistic regression for individual voting and voting gaps between ethnic and nativity groups

	Vote (all respondents)	
	M1 Asian nativity	M2 Hispanic nativity
*Individual covariates*		
Asian nativity (Ref.cat: 3^rd^ gen.^d^)		
1^st.^ gen. Asian	−0.66 (0.02)^a^	–
2^nd^ gen. Asian	−0.18 (0.03)^a^	–
Hispanic nativity (Ref.cat: 3^rd^ gen.^d^)		
1^st.^ gen. Hispanic	–	−0.25 (0.02)^a^
2^nd^ gen. Hispanic	–	−0.13 (0.02)^a^
Age	0.03 (0.00)^a^	0.03 (0.00)^a^
Gender (male)	−0.05 (0.01)^a^	−0.05 (0.01)^a^
Marital status (Ref.cat: divorced)		
Married	0.43 (0.01)^a^	0.42 (0.01)^a^
Single	0.23 (0.01)^a^	0.22 (0.01)^a^
Separated	−0.07 (0.02)^a^	−0.06 (0.02)^b^
Widowed	−0.07 (0.02)^a^	−0.08 (0.01)^a^
Education (Ref.cat: no/primary educ.)		
Secondary. educ.	0.60 (0.01)^a^	0.59 (0.01)^a^
Tertiary educ.	1.12 (0.01)^a^	1.10 (0.01)^a^
Labor force	0.17 (0.01)^a^	0.17 (0.01)^a^
Black	0.21 (0.01)^a^	0.21 (0.01)^a^
*State covariates*		
Political ideology	−0.01 (0.00)^a^	−0.02 (0.00)^a^
Organizational density	0.04 (0.02)^c^	0.05 (0.02)^c^
Unemployment	0.02 (0.01)^c^	0.02 (0.01)^b^
GDP (log)	0.12 (0.14)	0.12 (0.14)
Urbanization	−0.01 (0.00)	−0.01 (0.00)
Foreign-born share	−0.01 (0.00)	0.00 (0.00)
*State FEs*	*yes*	*yes*
*Year FEs*	*yes*	*yes*
AIC	254,189	259,089
N	216,315	220,598

Logistic regression coefficients (robust standard errors clustered by state in parentheses). All models include state and year fixed effects (states = 50, years = midterm elections 2002, 2006, 2010). Significance codes: 0.001 < ^a^, 0.01 < ^b^, 0.05 < ^c^

Apart from substantiating the theoretically assumed voting gaps between voter groups, the detailed results in Table 2 confirm that individuals with more resources in terms of experience (age) or human and social capital (high education, active labor force participation, married) are more likely to vote than young voters with low education or divorced individuals, for instance. The findings also corroborate the high political mobilization of black voters, as well as a female overrepresentation among voters.

### Differential effect of direct democracy across ethnic and nativity groups

**Fig. 1:**
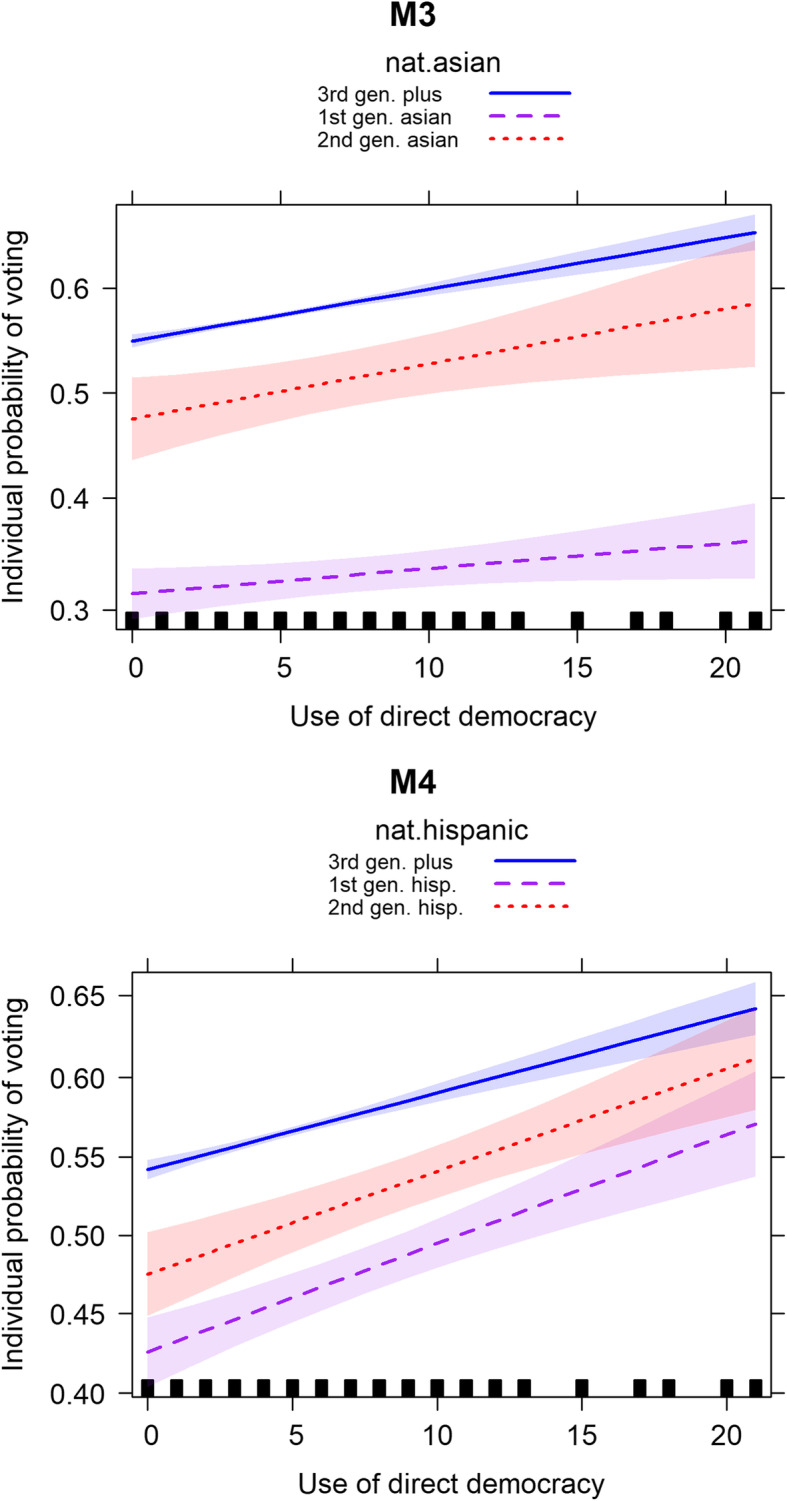
Predicted individual voting probabilities for different ethnic and nativity groups and use of direct democracy. Note: Predicted voting probabilities (lines) and 95% confidence intervals (bands) for interactions between ethnic and nativity groups and direct democracy based on Models 3–4 in Table OA2 in the online appendix

The next step of the analysis tests whether these voter gaps between ethnic groups and third-generation-plus voters are moderated by the frequent use of direct democracy, in line with theoretical expectations. To scrutinize the assumed heterogeneous effects, I complement the analyses with interaction terms between respondents’ ethnic background (first- and second generation Asian and Hispanic voters as compared to third-generation-plus voters) and the use of direct democracy (for detailed results see Table OA2 in the online appendix). The following discussion of the results focuses on the graphical illustration of these heterogeneous effects in terms of predicted probability plots (Fig. 1), as they allow for a substantive assessment and comparison of the effect sizes across different ethnic and nativity groups.

The graphs in Fig. 1 reveal characteristic and well documented voting patterns for individuals from certain ethnic nativity groups; they confirm, for instance, the pronounced voting gaps between first-generation Asian and third-generation-plus voters in Model 3 (Citrin and Highton 2002; Ramakrishnan and Espenshade 2001), or the higher voting propensity of second- compared to first-generation voters for both ethnic groups (Models 3 and 4). These varying starting points notwithstanding, individual voting propensities across all groups are higher in states that use direct democracy on average 20 times, compared to states with no use of direct democracy, in the 2 years preceding a midterm election, confirming the theoretical baseline assumption that frequent direct democratic elections activate voter turnout.

The increase in voting probabilities is considerable, amounting to plus 10 percentage points for third-generation-plus voters if they reside in a state with the most frequent use, compared to states with no use of direct democracy (Models 3 and 4 in Fig. 1). Relating this result to Hispanic voters, Model 4 shows that first-generation Hispanic voters, out of all ethnic and nativity groups, do indeed have the strongest reaction to frequent direct democratic elections, with an increase in voting probabilities of plus 15 percentage points. A look at Model 4 in Table OA2 in the online appendix confirms that this differential effect is significant, which means the voting gap between first-generation Hispanics and third-generation-plus voters can be reduced by 5 percentage points in states with frequent direct democratic elections, which is a considerable decrease. In other words, frequent use of direct democracy not only increases Hispanic voter turnout in absolute terms, similar to the other groups in Fig. 1, but it can even mitigate the lack of political integration, or the negative voting gap, between first-generation Hispanic and third-generation-plus voters, in line with theoretical expectations.

Turning to first-generation Asian voters, Model 3 confirms the theoretical expectation that frequent direct democratic elections only effect a small increase in voting probabilities among this group (plus 5 percentage points, as compared to plus 10 percentage points for third-generation-plus voters). Again, the results from Model 3 in Table OA2 in the online appendix reveal that this differential effect is significant. As theoretically expected, the negative voting gap between first-generation Asian and third-generation-plus voters widens by 5 percentage points in states with frequent direct democratic elections. Similar to the integrating pattern for first-generation Hispanics, this disintegrating pattern for first-generation Asian voters is of considerable size.

With respect to second-generation voters, the graphs for Models 3 and 4 in Fig. 1 show that the increase of voter probabilities among second-generation voters is slightly higher than for third-generation-plus voters (plus 11 percentage points for second-generation Asian voters, plus 14 percentage points for second-generation Hispanic voters). While second-generation Asian voters are only marginally and not significantly more mobilized via direct democracy than third-generation-plus voters, the higher coefficient for second-generation Hispanic voters aligns with theoretical expectations, yet it is not statistically significant.

As California is the state where direct democracy is used most frequently, and it is among the states with the highest shares of Asians and Hispanics among the population, I assess in a next step the extent to which the findings in Models 3 and 4 are influenced by California. Models 5 and 6 in Table OA2 show the respective results for Asian and Hispanic nativity groups and third-generation-plus voters excluding California from the sample, covering the remaining 49 states over the three midterm elections in 2002, 2006 and 2010. As the results of this conservative robustness test show, while the integrative effect for first-generation Hispanics is no longer significant based on this reduced sample, the disintegrating pattern remains significant for first-generation Asian voters.

### Does immigrant-related content of direct democratic elections matter?

In the next analytic steps, the robustness of the main findings will be further tested. So far, the study supports the institutional argument that the absolute quantity of direct democratic ballots influences voter turnout to a varying extent. A first robustness check pays closer attention to the nature of direct democratic propositions. Existing research reveals that specific ballots, e.g. on morally charges issues, have a stronger mobilizing effect than votes on other issues (Biggers 2014). Applying this argument to the context of this study, I test whether turnout among different ethnic nativity groups is mainly driven by immigrant-related direct democratic initiatives and referendums, when compared to third-generation-plus voters. To do so, I identified the subsample of immigrant-related direct democratic initiatives and referendums through the Ballot Measures Database of the National Conference of State Legislatures (NCSL).^7^ An example of an anti-immigrant ballot proposal is Proposition 300, adopted by Arizona in 2006; it limits public program eligibility (e.g. access to university tuition) to US citizens, legal residents or otherwise lawfully present individuals.

A closer inspection of the respective immigrant-specific ballots listed in Table OA3 in the online appendix shows that only 1.85% (25 out of 1354 referendums or initiatives put to state ballots between 2000 and 2010) relate to legal or constitutional matters concerning immigrants. This is in line with earlier research on direct democracy in the US which shows that initiatives are used most frequently for mundane issues such as health, education, and civil or constitutional matters (Donovan 2014), whereas only a small share of ballot propositions relate to racially targeted propositions (Hajnal et al. 2002). Table OA3 also confirms that if immigrant and ethnic-racial issues are at stake, direct democracy tends to be used in an anti-immigrant manner: 72% of all immigrant-related ballots had anti-minority content, and 80% of these ballots had an anti-minority outcome. Analyses based on this small number of immigrant-related referendums and initiatives yield no significant differential effect on voter turnout across ethnic and nativity groups (Table OA4). Given the rich research documenting mobilizing effects of specific anti-immigrant propositions on voters with an immigrant background, this null finding raises the question whether the number of immigrant-related direct democratic ballots is just too small to quantify differential effects on immigrants. More recent research on the effect of state legislation overall, not just the small subsample of legislation adopted via direct democratic votes, suggests that also the quantity of restrictive legislation matters for mobilization via threat. Filindra and Manatschal (2019) show for instance that the number of policies restricting immigrants’ access to social benefits significantly increases Hispanic voters’ turnout rates relative to White voters.

### Alternative measures of direct democracy and voting

Additional robustness tests further confirm that the differential effects of direct democracy are most pronounced when considering the effective use of direct democracy. Using an index on the mere institutional provision of direct democratic instruments in a state (index from 1 to 5) only alters the gap between first-generation Asian and third-generation-plus voters in the expected negative direction, but no longer reduces the gap between first-generation Hispanic and third-generation-plus voters (Table OA5 in the online appendix).^8^ Further analyses confirm the exceptional character of presidential elections, since the differential effect of direct democracy on first-generation Hispanic and Asian voters relative to third-generation-plus voters emerges only in midterm elections, not in presidential election years (Table OA6 in the online appendix).

### Causality concerns

A final set of robustness checks addresses causality concerns, as the correlational patterns reported in the empirical section may suffer from endogeneity in the form of reversed causality or unobserved variable bias. The argument that institutions are endogenous to political behavior has long been discussed in the literature (Coleman 1990). To address potential reverse causality, I apply an Instrumental Variable approach. I further assess whether self-selection into naturalization distorts the effects of direct democracy on first-generation voters using a Heckman selection model to account for unobserved variable bias. A detailed explanation and theoretical justification of these additional statistical tests is provided in Supplement OA1 in the online appendix. Table OA7 in the online appendix illustrates the results of the Instrumental Variable approach using the citizen voting age population (per 1 million citizens) as an instrument for the use of direct democracy to account for the exogenous effect of direct democracy on voter turnout. The instrumented effect of direct democracy on individual voting is still positive and statistically significant. The chosen instrument satisfies additional statistical tests: the first-stage coefficient for the use of direct democracy regressed on the citizen voting age population amounts to 0.24 (*p* < 0.001), and the Wald test of exogeneity confirms that the null hypothesis of no endogeneity for the instrumented variable “use of direct democracy” can be rejected (Prob. > chi^2^ = 0.10). Bearing in mind the limitations of the instrument used, I interpret this result as an indication that the effect does indeed run from the use of direct democracy toward voting, in line with the direction of my theoretical argument. Turning to the Heckman selection model, the results of the outcome model on first-generation voters show that direct democracy remains a significant predictor of first-generation voting, even when controlling for the inverted selection propensity (Inverse Mill’s Ratio) of naturalization (Table OA8 in the online appendix).

## Discussion

Direct democracy is known for its positive impact on political participation as much as for its majoritarian nature and occasionally blunt anti-immigrant outcomes. Citizens with an ethnic background, in turn, remain severely underrepresented in the politics of destination countries. At the same time, research on immigrant political mobilization in the US reveals important variance in terms of political mobilization across different ethnic and nativity groups.

Building on these different literatures, this study tests the (dis-)integrative potential of direct democracy on individual voter turnout depending on ethnic group affiliation and nativity status. To do so, I developed theoretical expectations around individual voters’ varying responsiveness to the activating effect of frequent direct democratic elections, depending on whether they belong to first- or second-generation Asian or Hispanic voters as compared to third-generation-plus voters. The study identified two group-specific factors that should influence individual responsiveness to direct democratic elections: average group familiarity with the democratic process of voting and the level of non-electoral group political mobilization in the destination country at the time of study.

The empirical results confirm the expectations on differential effects of frequent direct democratic elections for the first ethnic voter generation. In states with frequent use of direct democracy, the negative voting gap between first-generation Asian and third-generation-plus voters increases even further, pointing to a disintegrating effect, whereas this gap decreases for first-generation Hispanic voters, suggesting an integrating tendency. The disintegrating trend for first generation Asian voters remains even significant if we remove California from the sample, the state with the most frequent use of direct democracy and one of the states with the highest Asian and Hispanic voter shares. While I observe a similar – yet not significant – integrating tendency for second-generation Hispanics, frequent direct democratic elections do not seem to affect second-generation Asian voters differently from third-generation-plus voters. Additional robustness checks further show that the differential quantitative effects of direct democracy on voter turnout can only be observed during “normal” electoral times (i.e. midterm elections) and when accounting for all direct democratic elections, rather than for the subset of immigrant-specific propositions.

The results of this study refine existing arguments on the outcomes of direct democracy by revealing patterns for differential effects of frequent direct democratic elections on individual voter turnout, depending on ethnic group affiliation and immigration background. Overall, empirical evidence provides more support for the disintegrating expectation. In particular, the differential effects for first-generation Asian voters illustrate that direct democracy can further segregate the electorate if voters belonging to a particular group are, on average, less familiar with democratic elections, and have lower levels of non-electoral political mobilization than third-generation-plus voters. Paradoxically, and in spite of its integrative potential, direct democracy is therefore not a tool for overcoming unequal participation; in fact, it may aggravate this inequality even further. Turning to the differential effects on Hispanic voters, the results of this study point, at best, to a potential integrating effect of direct democracy on voters who belong to groups that are experienced with democratic elections, in periods of elevated group political mobilization in the non-electoral arena. At worst, they suggest no differential effect between first-generation Hispanic and third-generation-plus voters, as analyses excluding California from the sample seem to suggest. Overall, the contrasting findings highlight the necessity to go beyond simplistic assumptions of a uniform effect of political institutions across voting groups.

Future research should further scrutinize and refine the theoretical arguments developed and tested in this article. The middle-range character of these arguments requires that scholars interested in testing differential effects in other contexts always account for the time, place and responsiveness of diverse ethnic and nativity groups in order to learn more about the (dis-)integrative potential of political institutions. The present study was limited as it could only analyze one particular political institution (direct democracy) in one particular setting (the US), and focused on pertinent ethnic and nativity groups in this specific context: first- and second- generation Asian and Hispanic voters compared to third-generation-plus voters. Future research could account more fully for the heterogeneity and intersectionality of immigrant political mobilization based on a more comprehensive set of factors including race, gender and socioeconomic status. While this study could substantiate average differential effects for first-generation Asian voters, the analyses for first-generation Hispanic voters excluding California suggest that future research should pay more attention to the heterogeneous effects of political institutions across places. Future studies could also analyze how immigrant voters are informed about upcoming direct democratic elections. Considering that 96% of first-generation Hispanic voters in this study’s CPS samples come from a Spanish-speaking country, content analysis of minority-language media could reveal the extent to which Spanish-language broadcasting may increase Hispanic voters’ responsiveness to the activating effect of direct democratic elections. Conversely, linguistic heterogeneity is much more pronounced among Asian voters, complicating uniform US broadcasting in Asian country-of-origin languages. The largest foreign-born Asian group in this study comes from the Philippines – a country that is already multilingual – which only accounts for 19% of all foreign-born Asian voters. Finally, studies outside the US context would reveal more about the (dis-)integrative force of political institutions elsewhere. As the US results for Hispanic voters show, even in a country with exceptionally high levels of ethnic political mobilization, the integrative potential of direct democracy appears to be limited. It might well be that in contexts with lower political mobilization of ethnic immigrant groups, as is the case in most European countries, the disintegrative effect of political institutions emerges even more strongly.

The limitations of this study notwithstanding, the findings presented here challenge the notion of political institutions as being neutral, especially with regard to the potential exclusion of voters from ethnic minority groups. The study points to the need to go beyond immigrant-specific policies or institutions to explain the civic and political behavior of voters with an ethnic and/or immigrant background. The evidence presented here is a step in this direction.

## Supplementary Information

**Additional file 1: Figure OA1.** Average yearly use of direct democracy (initiatives and referendums) in US states. **Table OA1.** Variables, operationalization, and data sources. **Table OA2.** Interaction models for different ethnic and nativity groups. **Table OA3.** Direct democratic ballots on immigrants, 1999–2010. **Table OA4.** Immigrant-related direct democratic ballots. **Table OA5.** Direct democratic institutions. **Table OA6.** Presidential election years. **Supplement OA1.** Causality concerns. **Table OA7.** Instrumental variable approach. **Table OA8.** Heckman selection model for first generation immigrants.

## Data Availability

The CPS voting supplement survey data analysed during the current study is available in the IPUMS repository [https://cps.ipums.org/cps-action/samples]. Data on use and institutions of direct democracy in US states were kindly provided by Bernauer and Vatter (2019), and are available in the GitHub repository by Julian Bernauer [https://github.com/julianbernauer/powerdiffusion]. Data collected by the author on the immigrant-related content of direct democratic ballots are provided in Table OA3 in the online appendix. **Reference:** Bernauer, Julian, and Adrian Vatter. 2019. *Power Diffusion and Democracy. Institutions, Deliberation and Outcomes*. Cambridge: Cambridge University Press.

## Footnotes

1 In line with established categorizations, the first immigrant generation denotes foreign-born individuals who immigrated to the US, while the second generation refers to US-born individuals with at least one foreign-born parent (Koopmans et al. 2012). Throughout this study, I use the terms Hispanics and Latinos interchangeably to refer to individuals who were born in or have ancestries coming from a country in Latin America.

2 Unlike the other studies cited, Stadelmann-Steffen and Freitag (2011) find a u-shaped, rather than a linear, relationship between representative versus direct democracy and voluntary engagement.

3 The example of US President Barack Obama illustrates the exceptional nature of presidential elections for this particular study: President Obama, who is known to have received exceptionally strong electoral support from voters with an immigrant background (Zepeda-Millán 2017, 192), was first elected in 2008, which falls within the period covered by this study.

4 This classification of democratic systems is based on the Democracy Index by the Economist (https://infographics.economist.com/2019/DemocracyIndex/ [last accessed: 8 October 2020]). Similar shares are observed for other election years.

5 Other surveys such as the American National Election Survey (ANES) or the Cooperative Congressional Election Study (CCES) do not reach the level of CPS in terms of nationwide coverage and nativity status of the respondents, nor the election years covered. Smaller survey projects addressing the political behavior and attitudes of specific ethnic or racial minorities, such as the National Black Election Study, the National Asian American Survey, or the Latino National Survey, are in turn too restricted in their scope and coverage.

6 Using multilevel analyses instead of state and year fixed effects, or running the models without state controls, does not alter the main findings reported in this paper.

7 My search was based on the following comprehensive list of key words: immig, migr, alien, asylum, foreign, refugee, citizenship, noncitizen, non-citizen, illegal [in the context of human beings], English, national origin, ethnic and race.

8 Similar results emerge, if a dummy denoting states where initiatives are possible is used instead (not reported here).

## References

[CR1] Abrajano M, Hajnal ZL (2015). White backlash: Immigration, race, and American politics.

[CR2] Alba R, Nee V (2003). Remaking the American mainstream: Assimilation and contemporary immigration.

[CR3] Barber B (1984). Strong democracy: Participatory politics for a new age.

[CR4] Barreto, M. A., Manzano, S., Ramírez, R., & Rim, K. (2009). Mobilization, participation, and Solidaridad. Latino participation in the 2006 immigration protest rallies. *Urban Affairs Review*, *44*(5), 736–764.

[CR5] Bernauer J, Vatter A (2019). Power diffusion and democracy. Institutions, deliberation and outcomes.

[CR6] Biggers DR (2011). When ballot issues matter: Social issue ballot measures and their impact on turnout. Political Behavior.

[CR7] Biggers DR (2014). Morality at the ballot. Direct democracy and political engagement in the United States.

[CR8] Blais A (2014). Why is turnout so low in Switzerland? Comparing the attitudes of Swiss and German citizens towards electoral democracy. Swiss Political Science Review.

[CR9] Bloemraad I (2006). Becoming a citizen in the United States and Canada: Structured mobilization and immigrant political incorporation. Social Forces.

[CR10] Bloemraad I, Voss K, Lee T, Voss K, Bloemraad I (2011). The protests of 2006. What were they, how do we understand them, where do we go?. Rallying for immigrant rights. The fight for inclusion in 21st century America.

[CR11] Bowler S, Donovan T (2002). Democracy, institutions and attitudes about citizen influence on government. British Journal of Political Science.

[CR12] Bowler S, Donovan T (2004). Measuring the effect of direct democracy on state policy: Not all initiatives are created equal. State Politics and Policy Quarterly.

[CR13] Bowler S, Nicholson SP, Segura GM (2006). Earthquakes and aftershocks: Race, direct democracy, and partisan change. American Journal of Political Science.

[CR14] Brady HE, Verba S, Lehman Schlozman K (1995). Beyond SES: A resource model of participation. American Political Science Review.

[CR15] Castles S (2010). Understanding global migration: A social transformation perspective. Journal of Ethnic and Migration Studies.

[CR16] Childers M, Binder M (2012). Engaged by the initiative? How the use of citizen initiatives increases voter turnout. Political Research Quarterly.

[CR17] Childers M, Binder M (2016). The differential effecs of initiatives and referenda on voter turnout in the United States, 1890-2008. Chapman Law Review.

[CR18] Cho WKT (1999). Naturalization, socialization, participation: Immigrants and (non-)voting. Journal of Politics.

[CR19] Citrin, J., & Highton, B. (2002). *How race, ethnicity, and immigration shape the California electorate*. San Francisco: Public Policy Institute of California.

[CR20] Coleman JS (1990). Foundations of social theory.

[CR21] Dahl RA (1989). Democracy and its critics.

[CR22] DeSipio L (1996). Making citizens or good citizens? Naturalization as a predictor of organizational and electoral behavior among Latino immigrants. Hispanic Journal of Behavioral Sciences.

[CR23] Donovan T (2014). Direct democracy: Lessons from the United States. Political Insight.

[CR24] Dyck JJ, Seabrook NR (2010). Mobilized by direct democracy: Short-term versus long-term effects and the geography of turnout in ballot measure elections. Social Science Quarterly.

[CR25] Filindra, A., & Manatschal, A. (2019). Coping with a changing integration policy context: American state policies and their effects on immigrant political engagement. *Regional Studies, 54*(11), 1546-1557. 10.1080/00343404.2019.1610167.

[CR26] Freitag M (2006). Bowling the state back in: Political institutions and the creation of social capital. European Journal of Political Research.

[CR27] Freitag M, Stadelmann-Steffen I (2010). Stumbling block or stepping stone? The influence of direct democracy on individual participation in parliamentary elections. Electoral Studies.

[CR28] Garcia Bedolla L (2005). Fluid borders: Latino power, identity, and politics in Los Angeles.

[CR29] Geys B (2006). Explaining voter turnout: A review of aggregate-level research. Electoral Studies.

[CR30] Greenstein FI (1965). Children and politics.

[CR31] Hajnal ZL, Gerber ER, Louch H (2002). Minorities and direct legislation: Evidence from California ballot proposition elections. The Journal of Politics.

[CR32] Hero RE, Tolbert CJ (2004). Minority voices and citizen attitudes about government responsiveness in the American states: Do social and institutional context matter?. British Journal of Political Science.

[CR33] Huddy L, Khatib N (2007). American patriotism, national identity, and political involvement. American Journal of Political Science.

[CR34] Koopmans R, Michalowski I, Waibel S (2012). Citizenship rights for immigrants: National political processes and cross-national convergence in Western Europe, 1980-2008. American Journal of Sociology.

[CR35] Krogstad, J. M. (2016). 2016 electorate will be the most diverse in U.S. history. Retrieved from http://www.pewresearch.org/fact-tank/2016/02/03/2016-electorate-will-be-the-most-diverse-in-u-s-history/.

[CR36] Leighley JE, Vedlitz A (1999). Race, ethnicity, and political participation: Competing models and contrasting explanations. Journal of Politics.

[CR37] Li, R., & Jones, B. M. (2019). Why do immigrants participate in politics less than native-born citizens? A formative years explanation. *Journal of Race, Ethnicity, and Politics, 5*(1), 62-91. 10.1017/rep.2019.22.

[CR38] Lien P-t, Conway MM, Wong JS (2004). The politics of Asian Americans: Diversity and community.

[CR39] Logan JR, Darrah J, Oh S (2012). The impact of race and ethnicity, immigration, and political context on participation in American electoral politics. Social Forces.

[CR40] Lupia A, Matsusaka JG (2004). Direct democracy: New approaches to old questions. Annual Review of Political Science.

[CR41] Manatschal A, Bernauer J (2016). Consenting to exclude? Empirical patterns of democracy and immigrant integration policy. West European Politics.

[CR42] Masuoka N, Han H, Leung V, Zheng BQ (2018). Understanding the Asian American vote in the 2016 election. Journal of Race, Ethnicity and Politics.

[CR43] Matsusaka JG (2004). For the many or the few. The initiative, public policy and American democracy.

[CR44] Ong, P. M. (2011). Defensive naturalization and anti-immigrant sentiment: Chinese immigrants in three primate metropolises. *Asian American Policy Review, 21*, 39–55.

[CR45] Pantoja AD, Menjívar C, Magaña L (2008). The spring marches of 2006. Latinos, immigration, and political mobilization in the 21st century. American Behavioral Scientist.

[CR46] Pantoja AD, Ramirez R, Segura GM (2001). Citizens by choice, voters by necessity: Patterns in political mobilization by naturalized Latinos. Political Research Quarterly.

[CR47] Ramakrishnan K (2005). Democracy in immigrant America. Changing demographics and political participation.

[CR48] Ramakrishnan K, Espenshade TJ (2001). Immigrant incorporation and political participation in the U.S. International Migration Review.

[CR49] Ramirez R (2013). Mobilizing opportunities: The evolving Latino electorate and the future of American politics.

[CR50] Smith D, Tolbert C (2007). Educated by initiative: The effects of direct democracy on citizens and political organizations in the American states.

[CR51] Stadelmann-Steffen I, Freitag M (2011). Making civil society work: Models of democracy and their impact on civic engagement. Nonprofit and Voluntary Sector Quarterly.

[CR52] Vatter A, Stadelmann-Steffen I, Danaci D (2014). Who supports minority rights in popular votes? Empirical evidence from Switzerland. Electoral Studies.

[CR53] Verba S, Schlozman KL, Brady HE, Nie NH (1993). Race, ethnicity and political resources - participation in the United-States. British Journal of Political Science.

[CR54] Vernby K (2013). Inclusion and public policy: Evidence from Sweden’s introduction of noncitizen suffrage. American Journal of Political Science.

[CR55] Wass H, Blais A, Morin-Chassé A, Weide M (2015). Engaging immigrants? Examining the correlates of electoral participation among voters with migration backgrounds. Journal of Elections, Public Opinion and Parties.

[CR56] Wong JS, Ramakrishnan K, Lee T (2011). Asian American political participation: Emerging constituents and their political identities.

[CR57] Zepeda-Millán C (2017). Latino mass mobilization: Immigration, racialization, and activism.

