# Supplementary material for: The integrative force of political institutions? Direct democracy and voter turnout across ethnic and nativity groups
Source: Comp Migr Stud. 2021 Feb 22;9(1):6. doi: 10.1186/s40878-020-00216-y (PMC7900089; doi:10.1186/s40878-020-00216-y)
Supplement: Supplementary file 1 — Additional file 1: Figure OA1. Average yearly use of direct democracy (initiatives and referendums) in US states. Table OA1. Variables, operationalization, and data sources. Table OA2. Interaction models for different ethnic and nativity groups. Table OA3. Direct democratic ballots on immigrants, 1999–2010. Table OA4. Immigrant-related direct democratic ballots. Table OA5. Direct democratic institutions. Table OA6. Presidential election years. Supplement OA1. Causality concerns. Table OA7. Instrumental variable approach. Table OA8. Heckman selection model for first generation immigrants. [file 40878_2020_216_MOESM1_ESM.docx]

**The integrative force of political institutions? Direct democracy and voter turnout across ethnic and nativity groups**

# Online Appendix

**Figure OA1** *Average yearly use of direct democracy (initiatives and referendums) in US states*


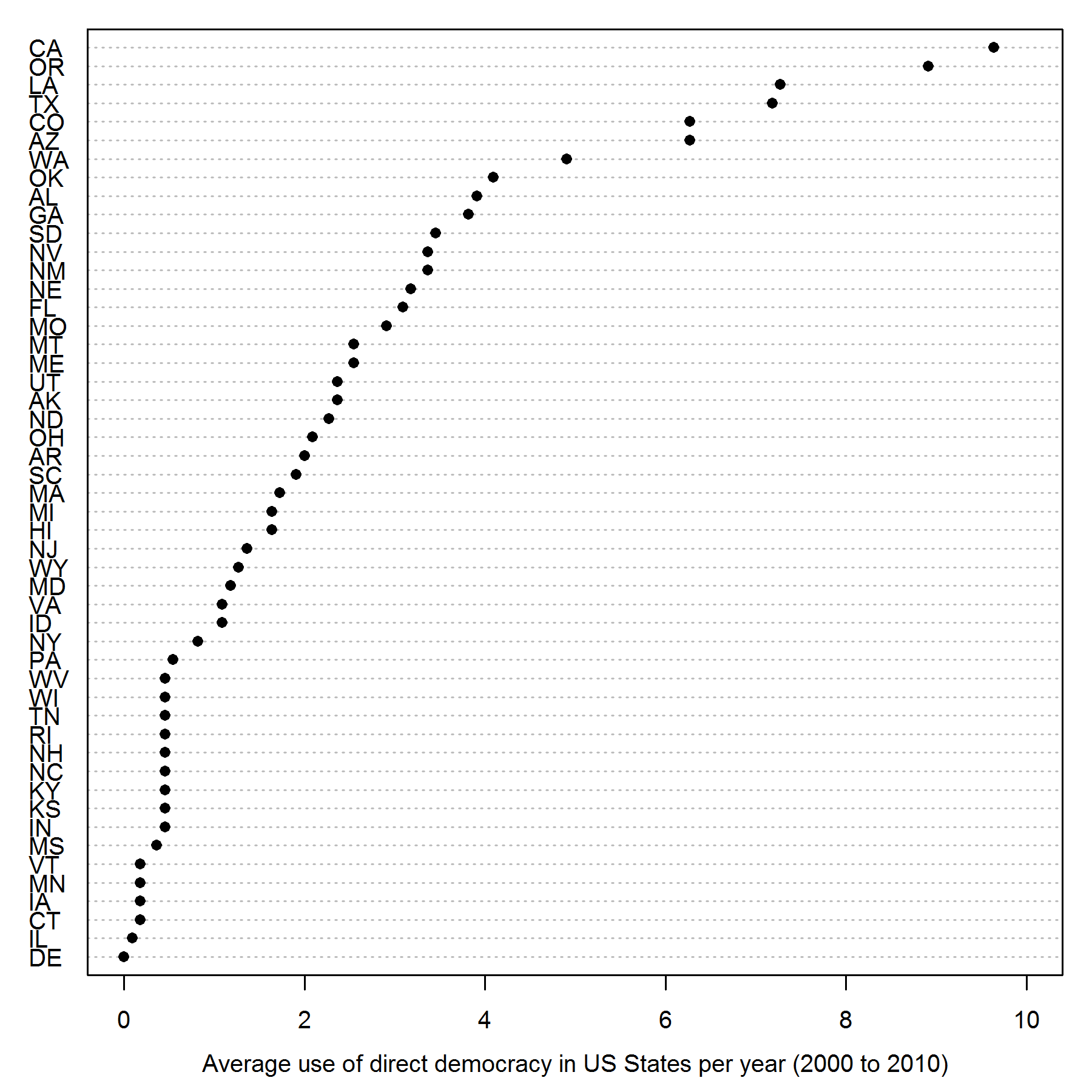


*Data*: Bernauer and Vatter (2019), own calculation.

**Table OA1** *Variables, operationalization, and data sources*

| Variable | Summary statistics | Operationalization/source* |
| --- | --- | --- |
| *Dependent variable* | | |
| Vote | *Shares* (voted): 55.7% | Dummy: 1 = voted, 0 = did not vote in most recent midterm election |
| *Individual covariates* | | |
| Asian nativity | *Shares*  3^rd^ gen. plus: 97.1%  1^st^ gen. Asian: 2.1%  2^nd^ gen. Asian: 0.8% | Categories: (1) 3^rd^ generation plus; (2) 1^st^ generation Asian; (3) 2^nd^ generation Asian |
| Hispanic nativity | *Shares*  3^rd^ gen. plus: 95.2%  1^st^ gen. Hisp.: 2.6%  2^nd^ gen. Hisp.: 2.2% | Categories: (1) 3^rd^ generation plus; (2) 1^st^ generation Hispanic; (3) 2^nd^ generation Hispanic |
| Age | Mean: 47.6  SD: 17.4  Min.: 18  Max: 85 | Age (in years) of respondent |
| Gender | *Share* (Male): 47% | Dummy: 1 = male, 0 = female |
| Black | *Share* (Black): 9.3% | Dummy: 1 = black, 0 = non-black |
| Marital status | *Shares*  Married: 58%  Separated: 2%  Divorced: 11%  Widowed: 7%  Single: 22% | Marital status of respondent, five categories: Married, separated, divorced, widowed, single |
| Education | *Shares*  Primary education: 11%  Secondary education: 52%  Tertiary education: 37% | Highest completed level of education, 3 categories: (1) no or primary education; (2) secondary education; (3) tertiary education |
| Labor force | *Share* (in labor force): 68% | Dummy: 1 = in labor force, 0 = not in labor force |
| Years of residence | Mean: 26.5  SD: 13.6  Min.: 0  Max.: 61 | Years since respondent immigrated in the United States |
| *State covariates* | | |
| Pol. ideology | Mean: 0.6  SD: 9.3  Min.: -41  Max: 22 | Relative measure of state party performance: Positive values = more republican, negative values = more democratic than the national Presidential election result  Source: Partisan Voter Index by State, 1994-2014 (Cook Political Report) |
| Org. density | Mean: 5.5  SD: 2.9  Min.: 2.9  Max.: 28.1 | Number of 501(c)3 organizations in a state per 1000 residents  Sources: National Center for Charitable Statistics, Population Division of the US Census Bureau, own calculation |
| Anti-immigrant attitudes | Mean: 44.7  SD: 9.7  Min.: 18.2  Max: 66.7 | Share of respondents per state indicating 4 or 5 to the question: Do you think the number of immigrants from foreign countries who are permitted to come to the United States to live should be: 1 = increased a lot; 2 = increased a little; 3 = left the same as it is now; 4 = decreased a little; 5 = decreased a lot  Source: American National Election Study (ANES) |
| Unemploy­ment | Mean: 5.5  SD: 1.5  Min.: 2.9  Max: 10.3 | Average unemployment rate per state in the four years preceding the midterm elections 2002, 2004, 2008  Source: US Department of Labor, Bureau of Labor Statistics |
| GDP | Mean: 46087.3  SD: 7591.3  Min.: 29223.7  Max: 68793.8 | Gross domestic product per capita (in USD) and state  Source: US Department of Commerce, Bureau of Economic Analysis |
| Urbaniza­tion | Mean: 75.5  SD: 14.6  Min.: 38.2  Max: 95 | Share of population living in an urban area  Source: Census 2000, 2010 (own calculation: mean for 2005) |
| Foreign born share | Mean: 10.5  SD: 7.6  Min.: 0.8  Max: 28.6 | Share of foreign born immigrants per state  Source: CPS voting supplements, own calculation |
| Voting age population | Mean: 7.9  SD: 8.7  Min.: 0.4  Max: 37.3 | Citizen voting age population of a state in 1 million citizens  Source: Census 2000, five-year American Community Survey (2005-2009, 2008-2012) |
| Use of direct democracy | Mean: 5.0  SD: 5.4  Min.: 0  Max.: 21 | Use frequency of the following direct democratic instruments in the two years preceding a midterm election: (1) statutory initiative; (2) constitutional initiative; (3) statutory legislative referendum; (4) constitutional legislative referendum; (5) popular referendum  Source: Bernauer and Vatter (2019), own calculation |
| Immigrant- related direct democratic ballots | Mean: -0.03  SD: 0.5  Min.: -5  Max.: 1 | Additive count index on immigrant-related content of direct democratic ballots in the four years preceding each midterm election. Coding: pro-immigrant content = 1, anti-immigrant content = -1, no immigration-related content = 0. Source : Own data collection of immigrant- and immigration-related direct democratic initiatives and referendums through the Ballot Measures Database of the National Conference of State Legislatures (NCSL) |
| Institutions of direct democracy | Mean: 2.7  SD: 1.8  Min.: 1  Max.: 5 | Index on the provision direct democratic instruments ranging from 1-5, accounting for the following direct democratic instruments: (1) statutory initiative; (2) constitutional initiative; (3) statutory legislative referendum; (4) constitutional legislative referendum; (5) popular referendum  Source: Bernauer and Vatter (2019), own calculation |
| Initiative state | *Share* (initiative states): 48.3% | Dummy: 1 = initiative state, 0 = no initiative state  Source: Bowler and Donovan (2004) |

* All individual variables stem from the Current Population Survey (CPS) voting supplements 2002, 2006, and 2010.

**Table OA2** *Interaction models for different ethnic and nativity groups*

|  | All US states | | Without California | |
| --- | --- | --- | --- | --- |
|  | M3  *Asian nativity* | M4  *Hispanic nativity* | M5  *Asian nativity* | M6  *Hispanic nativity* |
| *Individual covariates* |  |  |  |  |
| Group (Ref.cat.: 3^rd^ gen. +) |  |  |  |  |
| 1^st^ gen. Asian | -0.61 (0.03) *** | -- | -0.60 (0.03) *** | -- |
| 2^nd^ gen. Asian | -0.19 (0.05) *** | -- | -0.19 (0.05) ** | -- |
| 1^st^ gen. Hispanic | -- | -0.29 (0.03) *** | -- | -0.26 (0.03) *** |
| 2^nd^ gen. Hispanic | -- | -0.17 (0.03) *** | -- | -0.16 (0.04) *** |
| *State covariates* |  |  |  |  |
| Use of direct democracy | 0.01 (0.00) *** | 0.01 (0.00) *** | 0.01 (0.00) *** | 0.01 (0.00) *** |
| Group*use of DD interactions (Ref.: 3^rd^ gen. +*DD use) |  |  |  |  |
| **1^st^ gen. Asian*DD use** | **-0.01 (0.00) *** | **--** | **-0.01 (0.00)** ^+^ | **--** |
| **2^nd^ gen. Asian*DD use** | **0.00 (0.01)** | **--** | **0.00 (0.01)** | **--** |
| **1^st^ gen. Hispanic*DD use** | **--** | **0.01 (0.00) *** | **--** | **0.00 (0.00)** |
| **2^nd^ gen. Hispanic*DD use** | **--** | **0.01 (0.00)** | **--** | **0.00 (0.00)** |
| *Individual controls* | *yes* | *yes* | *yes* | *yes* |
| *State controls* | *yes* | *yes* | *yes* | *yes* |
| *State FEs* | *yes* | *yes* | *yes* | *yes* |
| *Year FEs* | *yes* | *yes* | *yes* | *yes* |
| AIC | 254110 | 258998 | 239884 | 243631 |
| N | 216315 | 220598 | 204109 | 207495 |

*Note*: Logistic regression coefficients (robust standard errors clustered by state in parentheses). All models include state and year fixed effects (states = 50, years = midterm elections 2002, 2006, 2010). Significance codes: 0.001 < ***, 0.01 < **, 0.05 < *, 0.1 < ^+^. Control variables included in the analysis but not reported in the table for the sake of clarity include age, gender, marital status, education, labor force participation and race (Black) at the individual level, and political ideology, organizational density, unemployment, GDP (log), urbanization, and foreign born share at the state level.

**Table OA3** *Direct democratic ballots on immigrants, 1999-2010*

| State | Year | Code | Type | Name | Target pop. | Yes votes (%) | Content (pro/anti immig.) | Outcome (pro/anti immig.) |
| --- | --- | --- | --- | --- | --- | --- | --- | --- |
| Alabama | 2004 | Proposed Amend. 2 | Leg. Ref. | Repeal Constitutional Provisions | Racial groups | 49.9 | pro | anti |
| Arizona | 2000 | Prop. 203 | Initiative | English language for Children in Public Schools | Non-English speakers | 63 | anti | anti |
| Arizona | 2004 | Prop. 200 | Initiative | Arizona Taxpayer and Citizen Protection Act | Illegal immig. | 55.6 | anti | anti |
| Arizona | 2006 | Prop. 300 | Leg. Ref. | Public programm eligibility | Illegal immig. | 71.4 | anti | anti |
| Arizona | 2006 | Prop. 102 | Leg. Ref. | Standing in civil actions | Illegal immig. | 74.2 | anti | anti |
| Arizona | 2006 | Prop. 103 | Leg. Ref. | English as the Official Language | Non-English speakers | 74 | anti | anti |
| Arizona | 2008 | Prop. 202 | Initiative | Prohibiting Employers from Intentionally or Knowingly Employing an Unauthorized Alien | Unauthor. alien | 40.8 | anti | pro |
| Arizona | 2010 | Prop. 107 | Leg. Ref. | Preferential Treatment or Discrimination Prohibition | Minorities (racial, ethnic, gender, color, national origin) | 59.9 | anti | anti |
| California | 2003 | Prop. 54 | Initiative | Classification by Race, Ethnicity, Color or National Origin | Racial, ethnic, non-national groups | 36.1 | pro | anti |
| Colorado | 2002 | Referendum E | Leg. Ref. | Cesar Chavez Legal Holiday | Cesar Chavez | 18.1 | pro | anti |
| Colorado | 2002 | Amendment 31 | Initiative | English Language Education | Non-English speakers | 44.6 | anti | pro |
| Colorado | 2006 | Referendum K | Leg. Ref. | Immigration lawsuit against federal government | Immigrants | 55.7 | anti | anti |
| Colorado | 2006 | Referendum H | Leg. Ref. | Limiting a state business tax income deduction | Illegal immig. | 50.7 | anti | anti |
| Colorado | 2008 | Amendment 46 | Initiative | Discrimination and Preferential Treatment by Governments | Minorities (racial, ethnic, gender, color, national origin) | 49.2 | anti | pro |
| Florida | 2008 | Amendment 1 | Leg. Ref. | Relating to Property Rights / Ineligible Aliens | Ineligible aliens | 47.9 | pro | anti |
| Massa. | 2002 | Question 2 | Initiative | English Language Education in Public Schools | Non-English speakers | 68 | anti | anti |
| Michigan | 2006 | Proposal 06-2 | Initiative | A Proposal to Amend the State Constitution to Ban Affirmative Action Programs… | Minorities (racial, ethnic, gender, color, national origin) | 57.9 | anti | anti |
| Missouri | 2008 | Constit. Amendment 1 | Leg. Ref. | English as the Official Language | non-English speakers | 85.8 | anti | anti |
| Nebraska | 2002 | Proposed amendment 1 | Leg. Ref. | A constitutional amendment to clarify English language requirements in schools | Non-English speakers | 43 | pro | anti |
| Nebraska | 2008 | Initiative 424 | Initiative | Affirmative action ban | Minorities (racial, ethnic, gender, color, national origin) | 57.5 | anti | anti |
| New Mexiko | 2002 | Const. Amendment 4 | Leg. Ref. | Repeal Prohibition on Aliens Owning Land | Aliens | 46 | pro | anti |
| New Mexiko | 2006 | Const. Amendment 1 | Leg. Ref. | Protection of Right to Own Property | Aliens | 69.9 | pro | pro |
| Oklahoma | 2010 | Question 751 | Leg. Ref. | Providing that the English Language is the Common and Unifying Language of Oklahoma | Non-English speakers | 75.5 | anti | anti |
| Oregon | 2008 | Measure 58 | Initiative | Prohibits teaching public school student in language other than English for more than two years | Non-English speakers | 43.6 | anti | pro |
| Utah | 2000 | Initiative A | Initiative | English as the Official Language of Utah | Non-English speakers | 67.2 | anti | anti |

*Note:* Ballots identified via the National Conference of State Legislatures (NCSL) for the period 2000-2010, using the following key words: immig, migr, alien, asylum, foreign, refugee, citizenship, noncitizen, non-citizen, illegal [in the context of human beings], English, national origin, ethnic, and race.

**Table OA4** *Immigrant-related direct democratic ballots*

|  | *Asian nativity* | *Hispanic nativity* |
| --- | --- | --- |
| *Individual covariates* |  |  |
| Group (Ref.cat.: 3^rd^ gen. +) |  |  |
| 1^st^ gen. Asian | -0.66 (0.02) *** | -- |
| 2^nd^ gen. Asian | -0.18 (0.03) *** | -- |
| 1^st^ gen. Hispanic | -- | -0.25 (0.02) *** |
| 2^nd^ gen. Hispanic | -- | -0.13 (0.02) *** |
| *State covariates* |  |  |
| Immigrant-related DD content | -0.02 (0.01) *** | -0.02 (0.01) *** |
| Group*DD content interactions (Ref.: 3^rd^ gen. +*DD content) |  |  |
| 1^st^ gen. Asian*DD content | -0.04 (0.04) | -- |
| 2^nd^ gen. Asian*DD content | -0.02 (0.07) | -- |
| 1^st^ gen. Hispanic*DD content | -- | 0.03 (0.03) |
| 2^nd^ gen. Hispanic*DD content | -- | 0.02 (0.03) |
| *Individual controls* | *yes* | *yes* |
| *State controls* | *yes* | *yes* |
| *State FEs* | *yes* | *yes* |
| *Year FEs* | *yes* | *yes* |
| AIC | 254184 | 259086 |
| N | 216315 | 220598 |

*Note*: Logistic regression coefficients (robust standard errors clustered by state in parentheses). All models include state and year fixed effects (states = 50, years = midterm elections 2002, 2006, 2010). Significance codes: 0.001 < ***, 0.01 < **, 0.05 < *. Immigrant related content is an additive count index. Positive values represent pro- and negative values anti-immigrant content (see Tables OA1 and OA3). Control variables included in the analysis but not reported in the table for the sake of clarity include age, gender, marital status, education, labor force participation and race (Black) at the individual level, and political ideology, organizational density, unemployment, GDP (log), urbanization, and foreign born share at the state level.

**Table OA5** *Direct democratic institutions*

|  | *Asian nativity* | *Hispanic nativity* |
| --- | --- | --- |
| *Individual covariates* |  |  |
| Group (Ref.cat.: 3^rd^ gen. +) |  |  |
| 1^st^ gen. Asian | -0.58 (0.04) *** | -- |
| 2^nd^ gen. Asian | -0.23 (0.06) *** | -- |
| 1^st^ gen. Hispanic | -- | -0.26 (0.04) *** |
| 2^nd^ gen. Hispanic | -- | -0.15 (0.04) *** |
| *State covariates* |  |  |
| Direct democratic institutions | 0.05 (0.07) | 0.13 (0.07) ^+^ |
| Group*DD institution interactions (Ref.: 3^rd^ gen. +*DD institution) |  |  |
| 1^st^ gen. Asian*DD institution | -0.03 (0.01) * | -- |
| 2^nd^ gen. Asian*DD institution | 0.02 (0.02) | -- |
| 1^st^ gen. Hispanic*DD institution | -- | 0.00 (0.01) |
| 2^nd^ gen. Hispanic*DD institution | -- | 0.01 (0.01) |
| *Individual controls* | *yes* | *yes* |
| *State controls* | *yes* | *yes* |
| *State FEs* | *yes* | *yes* |
| *Year FEs* | *yes* | *yes* |
| AIC | 254186 | 259093 |
| N | 216315 | 220598 |

*Note*: Logistic regression coefficients (robust standard errors clustered by state in parentheses). All models include state and year fixed effects (states = 50, years = midterm elections 2002, 2006, 2010). Significance codes: 0.001 < ***, 0.01 < **, 0.05 < *, 0.1 < ^+^. Control variables included in the analysis but not reported in the table for the sake of clarity include age, gender, marital status, education, labor force participation and race (Black) at the individual level, and political ideology, organizational density, unemployment, GDP (log), urbanization, and foreign born share at the state level.

**Table OA6** *Presidential election years*

|  | *Asian nativity* | *Hispanic nativity* |
| --- | --- | --- |
| *Individual covariates* |  |  |
| Group (Ref.cat.: 3^rd^ gen. +) |  |  |
| 1^st^ gen. Asian | -0.72 (0.03) *** | -- |
| 2^nd^ gen. Asian | -0.24 (0.05) *** | -- |
| 1^st^ gen. Hispanic | -- | -0.24 (0.03) *** |
| 2^nd^ gen. Hispanic | -- | -0.09 (0.03) ** |
| *State covariates* |  |  |
| Use of DD | 0.00 (0.00) | 0.00 (0.00) |
| Group*use of DD interactions (Ref.: 3^rd^ gen. +*DD use) |  |  |
| 1^st^ gen. Asian*DD use | 0.00 (0.00) | -- |
| 2^nd^ gen. Asian*DD use | 0.00 (0.00) | -- |
| 1^st^ gen. Hispanic*DD use | -- | 0.00 (0.00) |
| 2^nd^ gen. Hispanic*DD use | -- | 0.00 (0.00) |
| *Individual controls* | *yes* | *yes* |
| *State controls* | *yes* | *yes* |
| *State FEs* | *yes* | *yes* |
| *Year FEs* | *yes* | *yes* |
| AIC | 212414 | 216714 |
| N | 207417 | 211207 |

*Note*: Logistic regression coefficients (robust standard errors clustered by state in parentheses). All models include state and year fixed effects (states = 50, years = presidential elections 2000, 2004, 2008). Significance codes: 0.001 < ***, 0.01 < **, 0.05 < *, 0.1 < ^+^. Control variables included in the analysis but not reported in the table for the sake of clarity include age, gender, marital status, education, labor force participation and race (Black) at the individual level, and political ideology, organizational density, unemployment, GDP (log), urbanization, and foreign born share at the state level.

**Supplement OA1**

**Causality concerns**

Observational analyses like the ones presented in this paper face inferential challenges in terms of endogeneity such as reversed causality or unobserved variable bias. The argument that institutions are endogenous to political behavior has long been discussed in the literature (Coleman 1990). A very common approach to address endogeneity challenges is to resort to instrumental variables. A valid instrument fulfills two conditions (Angrist and Krueger 2001, Dunning 2008): First, it is related to the endogeneous regressor “use of direct democracy”, but independent of the error term in the equation on direct democracy and voting (exogeneity assumption). Second, an instrument should be related to the independent variable of interest, whereas it should not be related to the dependent variable other than through the independent variable (exclusion restriction). I use the citizen voting age population of a state (per 1 Million citizens) as an instrument to estimate the exogenous effect of the use of direct democracy on voting. As Bowler and Glazer suggest (2008, 162), states with a larger voting age population have more human resources to undertake policy initiatives. They also tend to have more numerous and diverse interests seeking representation. This diversity makes it more difficult for governments to address these contrasting needs, which would imply that citizens seek other channels such as the ones provided by direct democracy to find representation for their interests (Bowler and Glazer 2008, 162, Tolbert 2003). What is more, whether initiatives or popular referendums result in actual popular votes hinges on the capability to collect enough signatures, which is expected to be easier in regions with a large voting age population (Bauer and Fatke 2014, Ladner 2002). A look at the data confirms this relationship: the size of the voting age population per million citizens is clearly and positively correlated with the use of direct democracy in a state (r = 0.59, p < 0.01). In line with the exogeneity assumption, I further expect that there is no unobserved confounder influencing both, the instrument voting age population and the error term in the equation on direct democracy and voting. I also assume that the size of the voting population affects voter turnout solely through the use of direct democracy, conditional on different control variables (exclusion restriction). This assumption could be challenged by the classical argument that the smaller the polity, the more citizens will act responsibly and competently in controlling public decisions (Dahl and Tufte 1973), which could reflect in higher turnout in smaller polities. However, recent empirical evidence confirms a negative relationship between population size and voter turnout only at the local level of municipalities (Gaardsted Frandsen 2002, Oliver 2000, van Houwelingen 2017), but it fails to do so at higher levels of government (Cancela and Geys 2016, Zabler, Reclam, and Grohs 2020). Accordingly, I consider the voting population at the state level a valid instrument, which should relate to voter turnout solely via the frequency of direct democratic ballots. State population size by itself should further not systematically relate to voter registration, given that voter registration laws are more uniform and registration costs lower than ever during the period under study here (Highton 2004).^[[1]](#footnote-1)^

A second inferential challenge regards the self-selection of first generation voters. Unlike immigrants from the second generation, who are US citizens by birth on US soil and therefore automatically eligible to vote once they are 18 years old, first generation immigrants must naturalize in order to be able to vote. The sample of first generation voters is thus censored, because we can only observe who votes or does not vote among naturalized individuals, but we do not have this information for individuals of the first generation who did not naturalize (Heckman 1976). The decision to become a US citizen implies a self-selection of a specific group, for instance particularly interested, socially connected or highly skilled immigrants, into the group of first generation voters. I will use Heckman selection models to account for this self-selection of first generation voters into naturalization, and to test the robustness of my findings. I use the length of residence in the US as an additional variable to explain the decision to naturalize in the selection model.^[[2]](#footnote-2)^ What the Heckman selection model cannot, and does not intend to do, is to account for the exact motives underlying naturalization decisions, which are varied and can range from political (e.g. possibility to vote) to more instrumental motivations relating to access to rights and benefits (Bloemraad 2006, Cho 1999, DeSipio 1996, Pantoja, Ramirez, and Segura 2001).

**Table OA7** *Instrumental variable approach*

|  |  |
| --- | --- |
| *State covariates* |  |
| Use of direct democracy (instrumented) | 0.02 (0.01) *** |
| *Individual controls* | *yes* |
| *State controls* | *yes* |
| *State FEs* | *yes* |
| *Year FEs* | *yes* |
| AIC | 1350222 |
| N | 246270 |

*Note*: Instrumental Variable model (standard errors clustered by state in parentheses) with “use of direct democracy” instrumented by “voting age population per one million citizens”. Significance codes: 0.001 < ***. Control variables included in the analysis but not reported in the table for the sake of clarity include age, gender, marital status, education, labor force participation, ethnicity (Asian, Hispanic), immigrant background and race (Black) at the individual level, and political ideology, organizational density, unemployment, GDP (log), urbanization, and foreign born share at the state level.

**Table OA8** *Heckman selection model for first generation immigrants*

|  | *Selection Model*  Naturalized  (1^st^ gen. immigrants) | *Outcome Model*  Immigrant voting  (1^st^ gen. voters) |
| --- | --- | --- |
| *Individual covariates* |  |  |
| Years of residence | 0.06 (0.00) *** | -- |
| *State covariates* |  |  |
| Use of direct democracy | 0.01 (0.00) * | 0.01 (0.01) * |
| Inverse Mill’s Ratio | -- | -0.54 (0.04) *** |
| *Individual controls* | *yes* | *yes* |
| *State controls* | *yes* | *yes* |
| *State FEs* | *yes* | *yes* |
| *Year FEs* | *yes* | *yes* |
| AIC | 38187 | 17300 |
| N | 38276 | 13846 |

*Note*: Probit Heckman selection model on first generation immigrants (robust standard errors clustered by state in parentheses). Significance codes: 0.001 < ***, 0.05 < *. Control variables included in the analysis but not reported in the table for the sake of clarity include age, gender, marital status, education, labor force participation, ethnicity (Asian, Hispanic) and race (Black) at the individual level, and political ideology, organizational density, unemployment, GDP (log), urbanization, and foreign born share at the state level.

References

Angrist, Joshua, and Alan B. Krueger. 2001. "Instrumental variables and the search for identification: From supply and demand to natural experiments." *Journal of Economic Perspectives* 15:69-85.

Bauer, Paul C., and Matthias Fatke. 2014. "Direct Democracy and Political Trust: Enhancing Trust, Initiating Distrust-or Both?" *Swiss Political Science Review* 20 (1):49-69.

Bernauer, Julian, and Adrian Vatter. 2019. *Power Diffusion and Democracy. Institutions, Deliberation and Outcomes*. Cambridge: Cambridge University Press.

Bloemraad, Irene. 2006. *Becoming a Citizen: Incorporating Immigrants and Refugees in the United States and Canada*. Berkeley, Los Angeles: University of California Press.

Bowler, Shaun, and Todd Donovan. 2004. "Measuring the Effect of Direct Democracy on State Policy: Not All Initiatives Are Created Equal." *State Politics and Policy Quarterly* 4 (3):345-363.

Bowler, Shaun, and Amihai Glazer. 2008. *Direct Democracy's Impact on American Political Institutions*. New York: Palgrave Macmillan.

Cancela, João, and Benny Geys. 2016. "Explaining voter turnout: A meta-analysis of national and subnational elections." *Electoral Studies* 42:264-275.

Cho, Wendy K. Tam. 1999. "Naturalization, Socialization, Participation: Immigrants and (Non-)Voting." *Journal of Politics* 61 (4):1140-1155.

Coleman, James Samuel. 1990. *Foundations of Social Theory*. Cambridge: The Belknap Press of Harvard University Press.

Dahl, Robert A., and Edward R. Tufte. 1973. *Size and Democracy*. Stanford: Stanford University Press.

DeSipio, Louis. 1996. "Making Citizens or Good Citizens? Naturalization as a Predictor of Organizational and Electoral Behavior Among Latino Immigrants." *Hispanic Journal of Behavioral Sciences* 18 (2):194-213.

Dunning, Thad. 2008. "Model Specification in Instrumental-Variables Regression." *Political Analysis* 16:290–302.

Gaardsted Frandsen, Annie. 2002. "Size and electoral participation in local elections." *Environment and Planning C: Government and Policy* 20:853-869.

Heckman, James J. 1976. "The Common Structure of Statistical Models of Truncation, Sample Selection and Limited Dependent Variables and a Simple Estimator for Such Models." *Annals of Economic and Social Measurement* 5 (4):475-492.

Highton, Benjamin. 2004. "Voter Registration and Turnout in the United States." *Perspectives on Politics* 2 (3):507-515.

Ladner, Andreas. 2002. "Size and direct democracy at the local level: the case of Switzerland." *Environment and Planning C: Government and Policy* 20:813-828.

Oliver, Eric. 2000. "City Size and Civic Involvement in Metropolitan America." *American Political Science Review* 94 (2):361-373.

Pantoja, Adrian D., Ricardo Ramirez, and Gary M. Segura. 2001. "Citizens by Choice, Voters by Necessity: Patterns in Political Mobilization by Naturalized Latinos." *Political Research Quarterly* 54 (4):729-750.

Rytina, Nancy. 2008. The Timing of Naturalization in the United States through 1996:The Experience of the Legal Immigrant Cohorts of 1977 and 1982. In *Working Paper*: Homeland Security Office of Immigration Statistics.

Tolbert, Caroline J. 2003. "Direct Democracy and Institutional Realignment in the American States." *Political Science Quarterly* 118 (3):467-489.

van Houwelingen, Pepijn. 2017. "Political participation and municipal election size: A meta-study." *Local Government Studies* 43 (3):408-428.

Zabler, Steffen, Raphale Reclam, and Stephan Grohs. 2020. "Size and democracy revisited – Vom Einfluss der Kreisgröße auf das Wählerverhalten in Deutschland." *Politische Vierteljahresschrift* 61 (87-110).

1. Recent changes in voter registration regulations introduced by several US states after 2010, which are said to have curbed voting access for immigrant and racial minorities, occurred after the period covered in this study (see David Cole: “One person, one vote in America? The ideal is often not the practice”, March 4, 2016, *Washington Post*). [↑](#footnote-ref-1)
2. Although the probability to naturalize increases with time, it is important to note that naturalization is no perfectly linear process (Rytina 2008). [↑](#footnote-ref-2)
